# Automatically Enhanced OCT Scans of the Retina: A proof of concept study

**DOI:** 10.1038/s41598-020-64724-8

**Published:** 2020-05-08

**Authors:** Stefanos Apostolopoulos, Jazmín Salas, José L. P. Ordóñez, Shern Shiou Tan, Carlos Ciller, Andreas Ebneter, Martin Zinkernagel, Raphael Sznitman, Sebastian Wolf, Sandro De Zanet, Marion R. Munk

**Affiliations:** 1 RetinAI Medical AG, Bern, Switzerland; 2 Department of Ophthalmology, Inselspital, University Hospital, University of Bern, Bern, Switzerland; 3 ARTORG Center, University of Bern, Bern, Switzerland

**Keywords:** Medical imaging, Software, Optical techniques

## Abstract

In this work we evaluated a postprocessing, customized automatic retinal OCT B-scan enhancement software for noise reduction, contrast enhancement and improved depth quality applicable to Heidelberg Engineering Spectralis OCT devices. A trained deep neural network was used to process images from an OCT dataset with ground truth biomarker gradings. Performance was assessed by the evaluation of two expert graders who evaluated image quality for B-scan with a clear preference for enhanced over original images. Objective measures such as SNR and noise estimation showed a significant improvement in quality. Presence grading of seven biomarkers IRF, SRF, ERM, Drusen, RPD, GA and iRORA resulted in similar intergrader agreement. Intergrader agreement was also compared with improvement in IRF and RPD, and disagreement in high variance biomarkers such as GA and iRORA.

## Introduction

OCT is a non-invasive, micrometer-resolution imaging technique that has found wide application in the diagnosis of corneal and retinal pathologies. Thanks to advances in electronics, precision optics and signal processing, OCT technology has steadily improved in image quality, speed and resolution. However, speckle noise and signal loss in deeper tissue remains a major limitation. Speckle noise is caused by a complex combination of thermal, electrical, multiple-scattering effects, as well as digital processing algorithms. Indeed, in retinal imaging, it is common to consider up to 75% of the pixel values as noise^1,2^.

**Figure 1:**
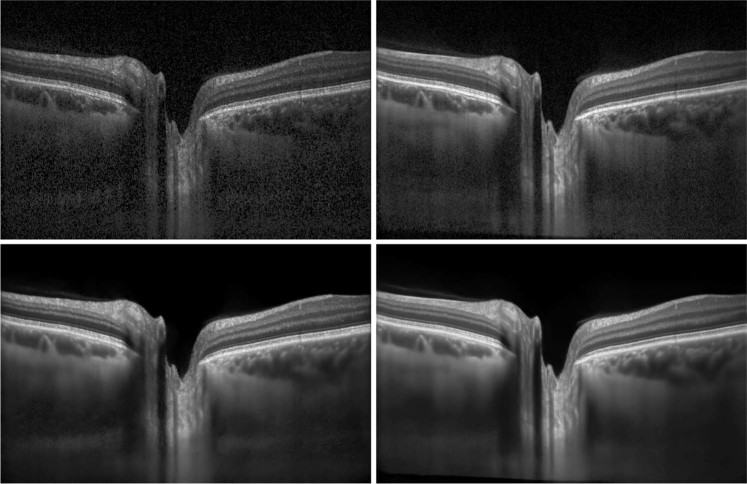
Example of images enhanced with averaging and the proposed algorithm. top left: original image from device; bottom left: enhanced image; top right: averaged image; bottom right: enhanced averaged image.

A common approach to improving OCT image quality is to acquire and average multiple scans of the same location. Assuming that noise is uncorrelated between the acquired images, the average of *N* images will improve the signal-to-noise by a factor of *N* while correlated noise will reduce the improvement in practice. Consequently, the approach requires a longer acquisition time, by a factor of *N*, during which the patient is required to fixate motionless on a fixation target. While this approach helps to improve images of patients with clear media, it results in rather unsatisfactory results in patients with media opacities e.g. cataracts. To mitigate this, commercial OCT devices often include a separate optical eye tracking system to support the process, with corresponding increases in cost and device complexity. Imperfections in patient fixation and the eye tracking system lead to blurriness in the averaged scans. Combined, the above create a practical ceiling to the image quality improvement that can be extracted from image averaging. See Fig. 1 for denoising and averaging examples.

Traditionally, digital noise removal attempts to post-process acquired images to reduce the amount of speckle noise without harming the structural information presence in the images sample. We identify two main areas in which OCT denoising has been evaluated, the first one considers spatial denoising methods, where image enhancement happens either via local image filtering such as median^3^ or mean Gaussian filters^4^, or at global OCT volume scale. The latter includes Block Matching 3D (BM3D)^5^, and later on with the application to the OCT field^6^ (in this case, to image human skin) as one of the most important contributions. Other spatial denoising methods consider the use of wavelets^7^, Bayesian optimization^8^, or diffusion filtering^9^. One more recent work considers using total variation diffusion combined with K-SVD for OCT volume denoising^10^. The key advantage of the aforementioned methods is their adaptability to multiple environments and scanning protocols.

More recently, deep convolutional neural networks have shown promising results in image enhancement. Strategies in spatial denoising considered using machine learning to target specific speckle noise distributions^11^, but the results were limited by the need for large amounts of data to train neural networks. To solve this issue, Lethinen *et al*.^12^ proposed a solution that in natural images that corrupted observations can be used to clean signals by including additional noise (i.e. Gaussian or Poisson). The inherent nature of the random noise combined with speckle noise could enable the quality improvement of OCT volumes, even when they were corrupted with non-random noise, such is the case with the pure text examples over images they present. More recently, deep learning methods have been successfully applied on reducing speckle noise of OCT images^13–15^.

Among the main issues of those approaches is the extended acquisition time, increase in patient discomfort and cost for healthcare systems. When enhancing medical images, it is of the utmost importance to avoid altering information that may influence the diagnostic assessment of the physician, for example by introducing artifacts or removing or adding clinically-relevant information. It is also important that physicians are familiarized with the appearance of the enhanced images compared to the original images. This holds regardless of the source of the enhancement: image averaging, digital signal processing or improved hardware.

Palma *et al*.^16^ evaluated image contrast and color setting of retinal OCT scans to assess retinal structures and morphology. They found that contrast and background affected the evaluation, however no setting was superior for all investigated features. Similarly, Giannakaki-Zimmermann *et al*.^17^ show that manual measurements of choroidal thickness is influenced by device-specific image settings, and that one of the six tested settings yields the best results.

In this work, we assess a digital signal processing algorithm for OCT enhancement, based on deep convolutional neural networks, by conducting a biomarker grading study by two expert ophthalmologists. Our purpose is a first proof of principle study to demonstrate non-inferiority in terms of biomarker visibility between original Spectralis OCT scans and their enhanced versions and to assess potential subjective and objective quality improvement.

### Methods

We trained a convolutional neural network to transform low quality, noisy OCT slices into high quality scans with reduced noise and better contrast. For this task we selected a training set from multiple devices with scans at multiple averaging degrees. Details are described below.

### Datasets

#### Test Set

The dataset gathered in Kurmann *et al*.^18^ was used to assess the quality of the enhancing algorithm. It comprises 1002 curated B-scans from a set of 6 ×6 mm Heidelberg Engineering Spectralis volume OCT scans, consisting of 49 B-scans, 9 times averaged. Pathologies included diabetic retinopathy, diabetic macular edema and early, intermediate and advanced AMD. Their given dataset was annotated by 8 annotators for multiple morphological biomarkers to create an accurate and precise ground truth based on maximum consensus and majority vote. The proposed here evaluated algorithm was applied to all images to produce a second set of enhanced images of the same size. Thus, in total 2004 individual B-scans were assessed by the graders. The presence or absence of the following morphological parameters according to a prespecified grading protocol were assessed: IRF, SRF, ERM, Drusen, RPD, GA and iRORA, PED, HE, SCAR_FIB. Respective parameters were assessed by two masked graders and retina specialists, who were previously trained for grading of respective biomarkers according to a pre-specified grading protocol. The definition of each parameter can be found in Supplemental Table 1. In addition, a subjective image quality measure with values ranging from 1 to 10 was collected for each image. A lower value indicates poor image quality due to noise, blur or other artefacts and inferior grading conditions, while a higher value points towards high image quality facilitating grading.

#### Labelling

**Figure 2:**
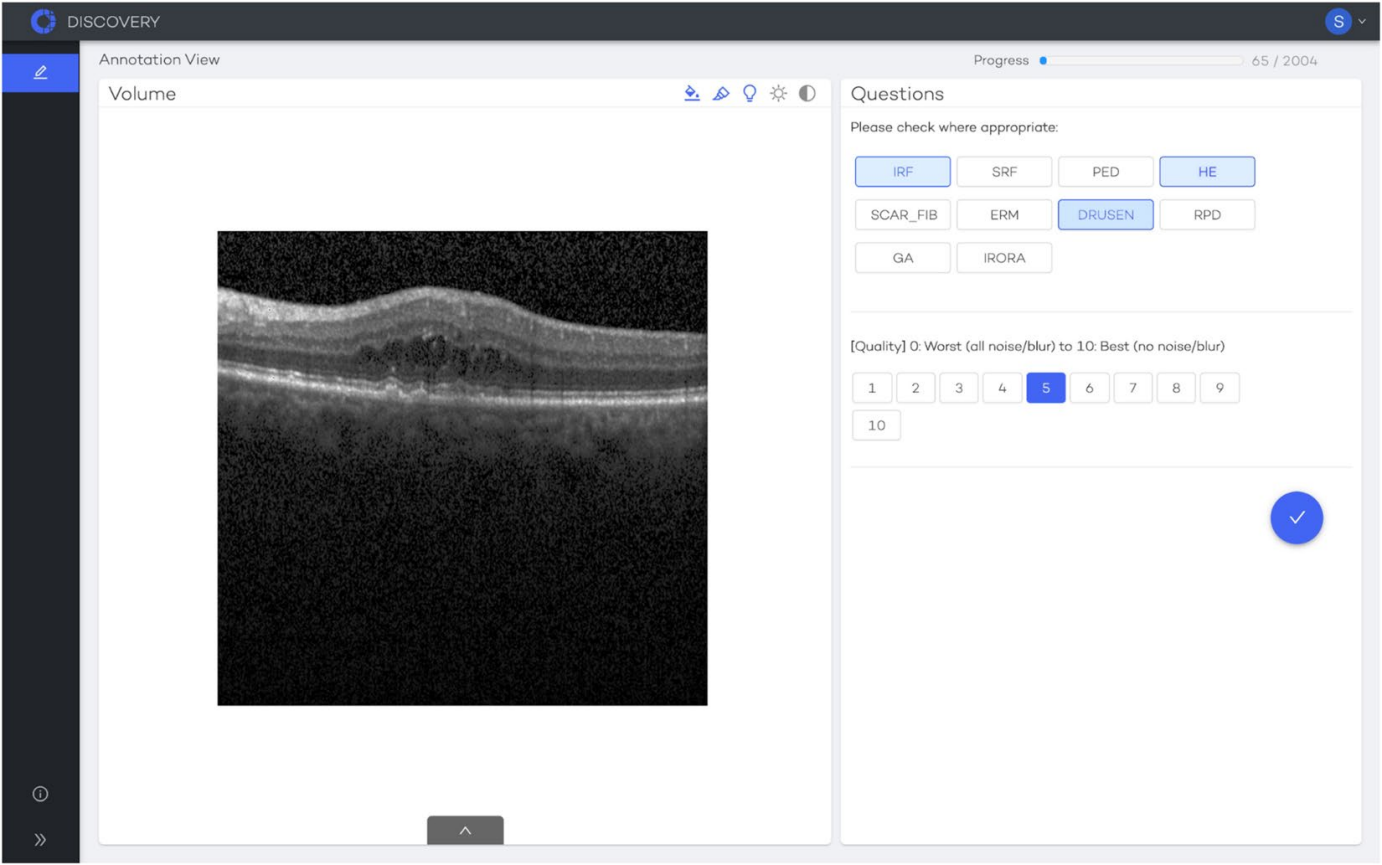
Example of RetinAI Discovery with an OCT slice and the grading interface in a browser.

Two masked expert graders labelled all 1002 original and corresponding 1002 enhanced B-scans in random order in a standardized dimmed environment using an equalized screen setting. To facilitate distributed labeling and ensure an independent random sampling of the images, the labelling tools of RetinAI Discovery were used (see Fig. 2). The B-scans were stored individually in the database along with the labelling questions. They were then presented to the graders in random order with RetinAI Discovery via the web. For authentication purposes, an account with individual login was created for each grader prior to the grading process. All 11 biomarkers were presented as non-exclusive options while image quality could be selected as a number from 1 to 10. Images were presented in random order to the masked graders, who were unaware which images was original or enhanced. Upon submission, the assessment of each individual B-scan of each grader were stored separately. At the end of the process, the results were downloaded from the database and then consolidated.

### Network

The enhancement network was a variation of the BRUNet from Apostolopoulos *et al*.^19^. This network accepts individual OCT B-scans as input; creates an image pyramid; applies a series of trained convolutional filters at different scales of the image pyramid, and outputs the enhanced image.

**Figure 3:**
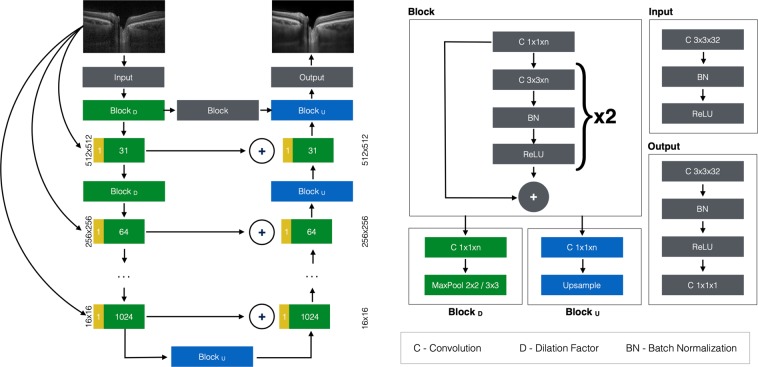
BRUNet version 9.4 architecture.

This version of BRUNet (Fig. 3), named 9.4, removes dilated convolutions and inception-style blocks in favour of two 3 × 3 back-to-back convolutional blocks per image pyramid level, on each of the downsampling and upsampling paths. Furthermore, it adds a horizontal convolutional block between the downsampling and upsampling blocks on the top-most pyramid level, and sums up (instead of concatenating) the output of every other downsampling block its corresponding block.

To train this network, a separate image training set was collected and used. This training set consisted of images from healthy volunteers using two commercial OCT devices (Spectralis OCT2, Heidelberg Engineering; Plex Elite 9000, Carl Zeiss Meditec) and one research device (Slit Lamp OCT, ARTORG Center, University of Bern^20^). A total of 5000 images were acquired, aligned and averaged to create the ground truth data. An additional synthetic training set was created by applying an ensemble of 72 neural networks, trained on synthetic images, on the same ground truth data. The training dataset was kept completely separate from the test set.

The synthetic training set was prepared as follows. First, we created three datasets, one per device. We then augmented those datasets with noise; noise + image intensity manipulation; noise + image intensity manipulation + geometric transformation. We applied two additive noise models separately: Gaussian noise with a standard deviation of 0.5 and Rayleigh noise with a sigma of 0.2. The image intensity adjustment consisted of brightness, contrast and gamma manipulation *ax*^g^ + *b*, with *a, b, g* ∈ [0.5, 1.5]. Finally, the geometric manipulation consisted of image rotation *r* ∈ [−45, 45] and image scale *s* ∈ [50%, 150%]. The parameters of the image intensity manipulation and geometric transformations were randomized for each training mini-batch.

We trained four neural network architectures on each of the datasets described above: standard U-net with transposed convolutions; standard U-net with bilinear upsampling; original BRUnet; and BRUnet 9.4. This resulted in 72 trained networks (4 architectures, 3 datasets, 2 noise models, 3 augmentations). We applied each of those networks on the training dataset and averaged their results to create the final synthetic training set. Total training time on four GPUs was in the order of three months.

Finally, four separate BRUNet 9.4 networks were trained and applied as an ensemble:

• trained on the synthetic training set only

• trained on the synthetic set and fine-tuned on the Slit Lamp OCT set

• trained on the synthetic set and fine-tuned on the Heidelberg Spectralis set

• trained on the synthetic set and fine-tuned on all ground truth data

Each of the four variations was trained to map a noisy input image to the corresponding averaged ground truth image. The input images were furthermore augmented with the same noise, image intensity and geometric manipulations that were applied during the generation of the synthetic training set. An additional Gaussian blur augmentation was applied on the input images with a standard deviation of 1.0. All augmentations were applied on the fly during training, with a 50% probability each. Training continued for 500 epochs using the *Adam* optimizer with a learning rate of 0.0001. The loss function was the sum of the MAE and the SSIM between the output of the network and the averaged ground truth image^21^.

**Figure 4:**
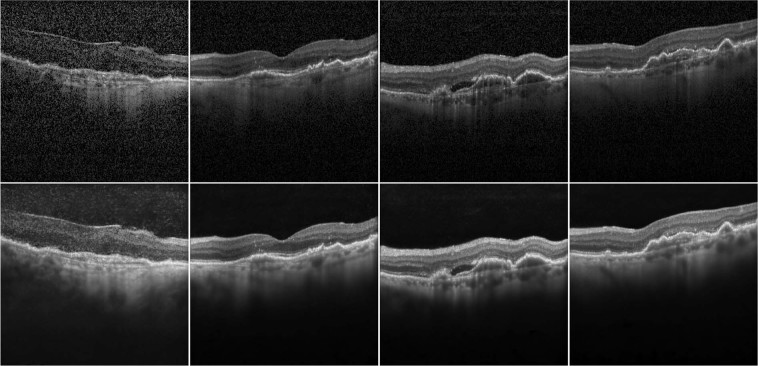
Example of original (top row) and enhanced images (bottom row). Subjective image quality increases from left to right. Original images were rated low quality mostly due to noise, absence of features, shadowing or blurriness. Highly rated images tend to show better contrast and lower noise levels, and often deeper penetration making the choroid more visible. In contrast, enhanced images show low to absent levels of noise in all images. In low quality cases, were the original image has an unusually low SNR, enhanced images tend to add blurriness or small artefacts. The contrast is higher than in original images, especially in the choroid.

During inference, the four trained networks were presented with two variations of the original OCT images: unmodified and histogram-equalized. The mean of the eight resulting images was stored and presented to the graders. Examples of original and enhanced scans can be seen in Fig. 4.

The median inference time for a single 512 × 512 B-scan was 240 per network on a common 4-core CPU (Intel i7-4770) and 66 on a common GPU (Nvidia GTX 1060). While this was not the goal of the experiment, real-time performance could be achieved for a single network by batch processing multiple B-scans at once.

### Evaluation method

Our evaluation is structured in three parts. First, we compare objective measures of image quality improvement between original and enhanced images. Second, the subjectively perceived image quality improvement using the semi-quantitative grading scale. Lastly, we evaluate the effect of enhancing on grading outcome for 7 biomarkers measured by intergrader agreement.

To objectively evaluate quality metrics, we used the algorithm presented by Immerkaer^4^, to estimate the noise level for each image of the dataset. This method is used to estimate the variance of additive zero mean Gaussian noise in an image. In addition, SNR was computed for both original and enhanced images. Due to a lack of background noise estimation, a window in the noisy area of the image was compared to a region with signal. Paired Student’s T-test was used to compare respective parameters pre- and post-processing.

The subjectively assessed quality assessment of the graders pre and post image enhancement was assessed using a Wilcoxon signed rank test.

Based on previous literature, Palma *et al*.^16^ and Giannakaki-Zimmermann *et al*.^17^, we assumed that the higher the level of *κ*, thus the higher the level of intergrader agreement of assessed parameters, the more reliable and the better was the OCT image. Given that we had 2 graders for this proof of principle study and that in such an analyses actually the individual graders and not the images are the dependent variable, we will show the intergrader agreement without performing inferential statistics for significant differences between kappa values.

## Experiments and Results

### Objective image quality assessment

**Figure 5:**
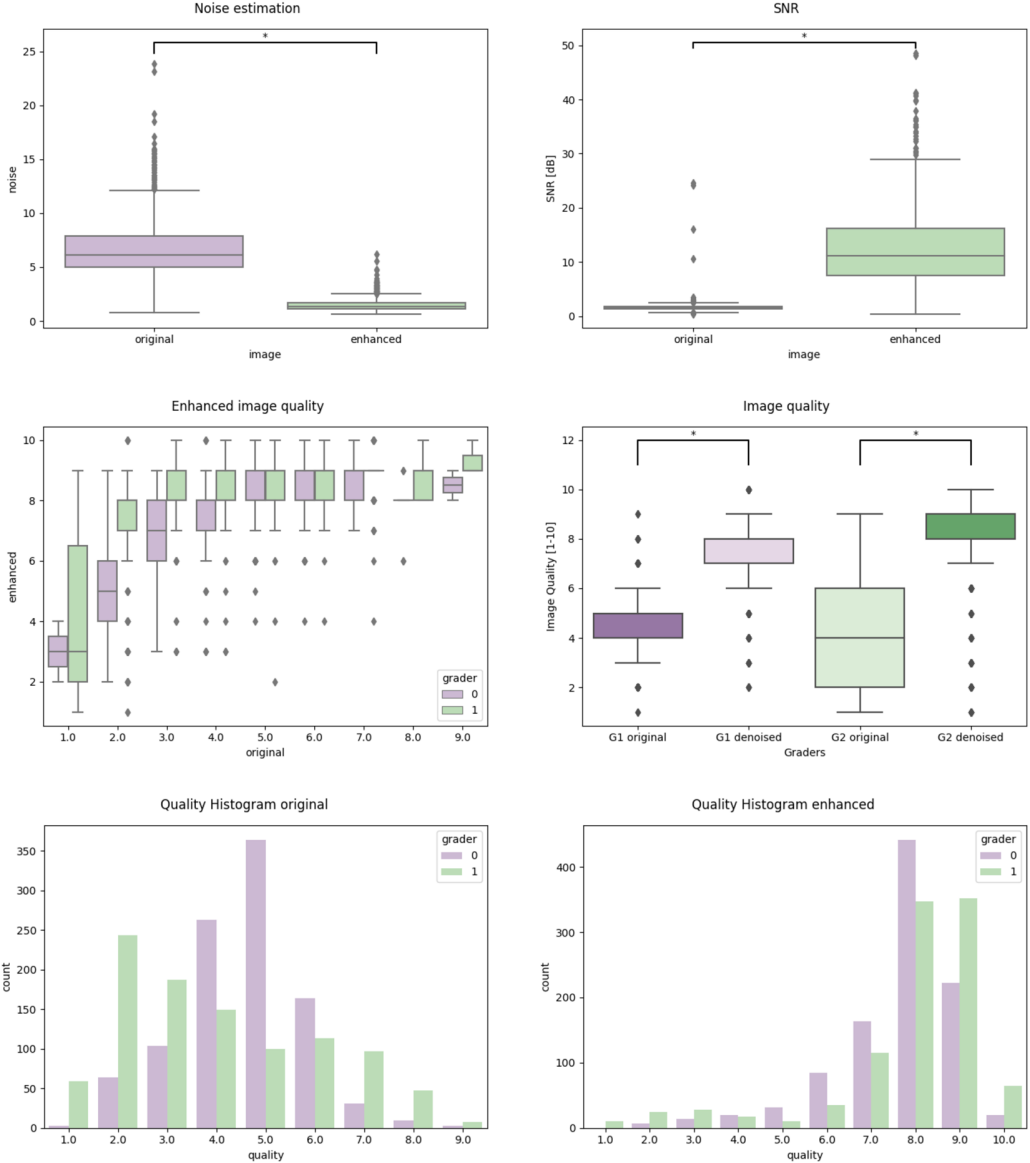
Image quality comparisons between original and enhanced images using SNR, estimated noise and subjective assessment.

As presented in Fig. 5a the noise levels were significantly higher (*p* < 0.01) in the original images compared to the enhanced, post processed images. Signal to noise ratio SNR significantly improved from 1.56 ± 0.46 to 12.32 ± 7.17 after post-processing (*p* < 0.01), see Fig. 5b.

### Subjective image quality

The image quality was assessed using a quality estimate ranging from 1 (very poor quality) to 10 (perfect quality). We compared the distribution of perceived image quality of each grader for the original vs. the enhanced images (see Fig. 5d). Significantly higher scores were achieved for the post processed, enhanced OCT images compared to the original images (*p* < 0.001) illustrates the improvement of perceived quality of up to 4 points in the enhanced images.

In order to evaluate how much the original subjective grading quality can be improved with our algorithm, we plotted the subjectively assessed image quality of the original vs. the enhanced images (Fig. 5c). As evident in the figure, very degraded images are hard to improve in quality. Between an initial quality score of 24, the quality can be improved up to 79, while an initial quality of 57 results in an increase of of 23 steps. Above a quality score of 7, there is a ceiling effect, and it is hard to significantly improve the already good quality score. Similarly, the distribution of quality across the full dataset clearly moves toward a higher score with a smaller variation. This can be also seen in the per-grader histograms in Fig. 5e,f.

### Assessment of morphological parameters

**Figure 6:**
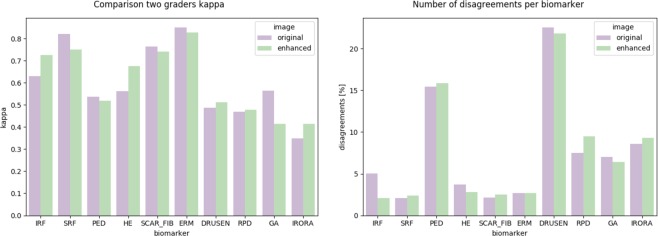
Grading agreement for original and enhanced images.

In the next step we evaluated the intergrader agreement of the assessed parameters in the original and the enhanced OCT image dataset, assuming that a higher intergrader agreement reflects better assessability and evaluation of the images. To assess intergrader agreement we evaluated Cohen’s Kappa values between the two graders of the original and enhanced images for each biomarker. Results can be seen in Fig. 6. Higher intergrader agreement of enhanced images can be observed for the parameters IRF, iRORA and HE, while intergrader agreement decreased for SRF and GA annotations after image enhancement. RPD, ERM, PED, SCAR_FIB and Drusen annotations showed similar results and were minimally affected by image enhancement.

## Discussion and Conclusion

In this paper we show that our proposed image enhancing algorithm significantly increases the image quality based on annotations from two graders on a large dataset of OCT B-scan slices. Graders prefer the enhanced images over original images, as it increases image quality both objectively and subjectively. Enhancing does not conclusively increase the agreement of the graders for all morphological parameters: A weak agreement reduction can be found for GA and to a lesser extent for SRF. While in SRF (*κ* = 0.82), there is still strong intergrader agreement (*κ* = 0.76), the Cohen’s Kappa for GA, which is mediocre for the original images (*κ* = 0.56), it decreases down to 0.41. This may be explained by the fact that an increase of contrast in the choroid due to enhancement, may be confused with higher transmission of light. Higher transmission is indicative of GA, as the light absorbing RPE is missing. This highlights an important point, that post processing of images may often lead to improvement in assessment of images, however it also involves the danger of misinterpretation. This phenomenon was also seen in a previous study on the impact of contrast on choroidal thickness assessment. Higher contrast on OCT led to significantly lower intergrader agreement in the measurement of choroidal thickness^17^. Interestingly the feature SRF was better identified using a high contrast setting in a previous study^16^. Fact is that our graders were used to assess original OCT images and although they consistently rated the enhanced images superior to the original in terms of quality, some parameters such as SRF and GA achieved higher intergrader agreement in the original compared to the enhanced images. A solution to this problem could be a preliminary training on enhanced images.

In contrast to GA, which is defined by increased choroidal transmission due to missing RPE, the precursor state iRORA, sometimes also called nascent GA, had stronger intergrader agreement in enhanced images vs. original images. iRORA is defined by some small areas of choroidal hypertransmission and RPE loss of less than 250 micrometers, not yet meeting the criteria for actual GA^22^. Enhancement of images seem benefit to detect these small patches of beginning GA. Also HE were easier to identify using enhanced images, which is consistent with previous observations^16^.

In general, images of low to mediocre quality profit most from this method, which is indicative of normalization to an acceptable level of image quality. The presented method only fails to clearly enhance images of very low original quality These are, in general, images which are the hardest to grade and assess. With more training data, we are confident that an improvement can be achieved also on these images. The algorithm faces a kind of â€œceilingâ€ effect in images with very high original quality, as it is hard to improve already clear images with very low SNR. Overall, normalization of image quality makes results more comparable and potentially lead to more consistent diagnosis.

This paper evaluates the effects of automatic image enhancement on image assessment by expert graders. It shows potential for image quality standardization, shorter acquisition times and thus higher patient comfort. Future, ongoing work includes a study with many graders to prove that not only quality but also grading performance and identification of morphological structures can be improved using respective software.

## Supplementary information

Supplementary Information.
