# Supplementary material for: Automatically Enhanced OCT Scans of the Retina: A proof of concept study
Source: Sci Rep. 2020 May 8;10:7819. doi: 10.1038/s41598-020-64724-8 (PMC7210925; doi:10.1038/s41598-020-64724-8)
Supplement: Supplementary file 1 — Supplementary Information. [file 41598_2020_64724_MOESM1_ESM.pdf]

Automatically Enhanced OCT Scans of the Retina: A proof of concept study

Stefanos Apostolopoulos[1], Jazmín Salas[2], José L. P. Ordóñez[2], Shern Shiou Tan[1], Carlos Ciller[1], Andreas Ebneter[2], Martin Zinkernagel[2], Raphael Sznitman[3], Sebastian Wolf[2], Sandro De Zanet[1,+], and Marion R. Munk[2,+,*]

[1]RetinAI Medical AG, Bern, Switzerland.
[2]Department of Ophthalmology, Inselspital, University Hospital, University of Bern, Bern, Switzerland.
[3]ARTORG Center, University of Bern, Bern, Switzerland.
[*]marion.munk@insel.ch
[+]these authors contributed equally to this work

Additional information

OCT Biomarker	Definition
Intraretinal Fluid	Oval well-defined hyporeflective areas with a minimal extension of 25 µm in any direction between the internal limited membrane and the photoreceptor layer and/or Diffuse darkening and thickening of the neurosensory retina.
Subretinal Fluid	Well-defined darkening with a minimal horizontal extension of 100 µm between the Retinal Pigment Epithelium (RPE) and photoreceptor layer.
Intraretinal Fluid	Oval well-defined hyporeflective areas with a minimal extension of 25 µm in any direction between the internal limited membrane and the photoreceptor layer and/or Diffuse darkening and thickening of the neurosensory retina.
Hard Exudates	Small points of increased reflectivity scattered throughout all retinal layers, primarily found in the near vicinity of intraretinal cystoid spaces. The size of HF can vary from 25 µm in diameter to 50 µm and they can be clustered.
Epiretinal Membrane	Thickening of the surface of the Retinal Nerve Fiber Layer (RNFL) within the whole macular cube scan. A hyperreflective line between the vitreomacular interface and the RNFL is visible.
Drusen	Located between the RPE and Bruch's membrane. They can appear hyper-reflective with some areas of hypo-reflectivity within the lesion depending on the composition. Bruch's membrane can be visible because the drusen is located between the RPE and Bruch's membrane. Drusen on the RPE can appear irregular, thinner and disrupted.
Reticular Pseudodrusen	Subretinal drusenoid deposits are seen in the subretinal space between the photoreceptors and the RPE. A typical sign of reticular pseudodrusen is an undulation of the ellipsoid zone. They are subretinal deposits, located above the RPE in contrast to traditional Drusen, which are located below the RPE.
Pigment Epithelium Detachmanet	Elevation of the RPE of more than 250 µm horizontal extent. The shape varies from well-demarcated/abrupt (serous) PED to an undulating appearance (drusenoid, fibrovascular). Reflectivity beneath the RPE may vary from homogeneously hyporeflective (serous) to moderate to hyperreflective (drusenoid/fibrovascular)
Fibrous Scar Tissue	Homogenous hyperreflective subretinal material.
incomplete Retinal Pigment Epithelium and Outer Retinal Atrophy	RPE thinning and loss of Ellipsoid Zone (EZ) and Interdigitation Zone (IZ) lines with more than 250 µm, which does not meet the criteria of Geographic Atrophy (GA) yet.
Geographic Atrophy	RPE thinning, loss of EZ and IZ lines, subsiding of the inner retinal layers as the outer layers are lost, and increased reflectivity of the Bruch's membrane and the choroid of at least 250 µm in diameter in any lateral dimension.

Table 1. Definitions of graded biomarkers.
